# Triclosan Enhances the Clearing of Pathogenic Intracellular *Salmonella* or *Candida albicans* but Disturbs the Intestinal Microbiota through mTOR-Independent Autophagy

**DOI:** 10.3389/fcimb.2018.00049

**Published:** 2018-02-21

**Authors:** Chao Wang, Zhongyang Yu, Xiaochen Shi, Xudong Tang, Yang Wang, Xueyan Wang, Yanan An, Shulin Li, Yan Li, Xuefei Wang, Wenjing Luan, Zhaobin Chen, Mingyuan Liu, Lu Yu

**Affiliations:** ^1^Key Laboratory of Zoonoses Research, Ministry of Education, Institute of Zoonosis, First Hospital of Jilin University, College of Veterinary Medicine, Jilin University, Changchun, China; ^2^Renmin Hospital of Wuhan University, Wuhan, China; ^3^Key Lab for New Drugs Research of TCM in Shenzhen, Research Institute of Tsinghua University in Shenzhen, Shenzhen, China; ^4^West China School of Public Health, Sichuan University, Chengdu, China; ^5^Shenzhen Nanshan Center for Disease Control and Prevention, Shenzhen, China; ^6^Jiangsu Co-innovation Center for Prevention and Control of Important Animal Infectious Diseases and Zoonoses, Yangzhou, China

**Keywords:** triclosan, autophagy, mTOR-independent, macrophage, antimicrobial

## Abstract

Triclosan (TCS) is a broad-spectrum antimicrobial agent, whose well-known antibacterial mechanism is inhibiting lipid synthesis. Autophagy, an innate immune response, is an intracellular process that delivers the cargo including pathogens to lysosomes for degradation. In this study, we first demonstrated that TCS induced autophagy in a dose-dependent manner in non-phagocytic cells (HeLa) and in macrophages (Raw264.7) and *in vivo*. The western blot results also revealed that TCS induced autophagy via the AMPK/ULK1 and JNK/ERK/p38 pathways independent of mTOR. The immunofluorescence results indicated that TCS up-regulated the expression of the ubiquitin receptors NDP52 and p62 and strengthened the co-localization of these receptors with *Salmonella enterica* Typhimurium (*S*. typhimurium) or *Candida albicans* (*C. albicans*) in infected MΦ cells. In addition, sub-lethal concentrations of TCS enhanced the clearing of the pathogens *S*. typhimurium or *C. albicans* in infected MΦ and in corresponding mouse infection models *in vivo*. Specifically, we found that a sub-inhibitory concentration of TCS induced autophagy, leading to an imbalance of the intestinal microflora in mice through the analysis of 16s rRNA Sequencing. Together, these results demonstrated that TCS induced autophagy, which enhanced the killing against pathogenic *S*. typhimurium or *C. albicans* within mammal cells but broke the balance of the intestinal microflora.

## Introduction

Triclosan (TCS) is a broad-spectrum anti-bacterial agent whose mechanism of action is reported to be blocking fatty acid synthesis (Gaulke et al., 2016). TCS is widely used in a large number of personal care products, household items, medical devices, and in clinical settings (Wu et al., 2017). However, there is a significant increase in hepatocellular adenomas and carcinomas in mice treated with TCS (Fang et al., 2010). A recent study also revealed that TCS induced mouse liver tumors by constitutively activating the androstane receptor (Yueh et al., 2014). The U.S. FDA issued that over-the-counter consumer antiseptic wash products containing TCS and certain active ingredients could no longer be marketed as of September 2, 2016 (U.S. FDA, 2016). Our knowledge is limited on the effects of TCS on human biology, but the relative interest is increasing.

As part of the innate immune response, autophagy plays an important role in the first line of defense against intruding pathogens. During this process, cargo including pathogens is delivered to a double-membrane structure called an autophagosome for degradation and recycling. The degradation is accomplished by the fusion of the autophagosome and lysosome (Wang et al., 2014). The ubiquitin-like conjugation systems, involving autophagy-related genes (ATG), participate in the classical signaling mechanism of autophagy.

Mammalian target of rapamycin (mTOR) is the key regulator of cell growth, metabolism, survival and the lifespan of organisms, and therefore, it is closely in line with autophagy. mTOR is a negative regulator of autophagy, while AMP-activated protein kinase (AMPK) is a positive regulator. ULK1 is the most upstream autophagy inducer, it plays a role in initiating the autophagy process, and it is directly regulated by mTOR or AMPK. Recent studies show that the extracellular signal-regulated kinase 1/2 (ERK1/2) pathway is also involved in regulating autophagy, and the c-jun N-terminal kinase (JNK) and p38 kinase simultaneously participate in this process (Bhui et al., 2010). A ubiquitin-dependent process is one route in the autophagic capture of bacteria in which the bacteria are surrounded by a coat of polyubiquitin, and the ubiquitinated coat is bound by ubiquitin interacting adaptor proteins such as p62, NDP52, NBR1, LRSAM1, etc (Cemma et al., 2011). Noteworthy, the upregulation of mTOR (Memmott and Dennis, 2010) or p62 (Duran et al., 2008) is reported to be related to the carcinogenic tendency of mammalian cells.

Macrophages (MΦ) are key members of the innate immune system, providing the first response to infection with various mechanisms, including autophagy. *Salmonella* typhimurium (*S*. typhimurium) can invade, survive, and replicate in phagocytic and non-phagocytic cells. However *S*. typhimurium could be selected for autophagy, relying on the accumulation of ubiquitin or autophagy adaptor proteins around the target bacteria (Cemma et al., 2011). Recent research demonstrates that macrophage autophagy kills the pathogenic fungus *C. albicans* (Nicola et al., 2012), but to our knowledge, there are no reports on how ubiquitin adaptor proteins work in autophagy against killing *C. albicans*.

The number of intestinal microflora is large, and the variety is broadly involved in various types of physiological activities. The steady state of the intestinal flora is vital to human health. Intestinal microflora, through a series of physiological effects, affect the intestinal mucosal immune function of dynamic regulation (Clemente et al., 2012). However, the study of TCS on intestinal flora is very limited and contradictory (Poole et al., 2016).

Therefore, this study reveals the relationship among TCS, autophagy and non-phagocytic cells/macrophages; explores the novel mechanisms of TCS-induced autophagy in anti-bacterium; and uncovers the effect of TCS treatment on the intestinal flora balance.

## Materials and methods

### Antibodies, chemicals, plasmids, and strains

The antibodies used in the experiments were purchased from Cell Signaling Technology (Maryland, USA), the triclosan (TCS) came from Sigma (Missouri, USA), and the other reagents, unless stated otherwise, were all purchased from Sigma (Missouri, USA). The pmRFP-LC3 is mammalian expression of rat LC3 fused to mRFP, the ptfLC3 is mammalian expression of rat LC3 fused to mRFP and EGFP, and the pEGFP-LC3 is mammalian expression of rat LC3 fused to EGFP (Kimura et al., 2007). *Lactobacillus, Escherichia coli* (*E. coli*), and *Enterococcus faecalis* (*E. faecalis*) were isolated from normal mouse feces. *S*. typhimurium ATCC 14028 and *C. albicans* SC5314 were kept by our laboratory.

### Determination of bacterial minimal inhibitory concentrations (MICs) and minimum bactericidal concentrations (MBCs)

The MICs were determined using the microdilution method as described previously (Moore et al., 2008). The MIC was defined as the lowest concentration for which bacterial growth did not occur. The MBC was defined as the lowest concentration of microbicide at which no growth occurred after 4 days of incubation.

### Antifungal susceptibility testing

The MICs of TCS against the *C. albicans* strains were determined by broth microdilution using two-fold serial dilutions in RPMI 1640 medium, as described by the CLSI method M27-A. The test was performed in 96-well, flat-bottomed microtitration plates according to previous studies (Sun et al., 2008).

### Cells and cell cultures

Intestinal epithelial cells and intestinal macrophages were extracted according to previous studies (Macartney et al., 2000; Yu et al., 2011). Raw264.7 cells and HeLa cells used in our study came from ATCC (Maryland, USA) and were maintained in DMEM supplemented with 10% fetal bovine serum (FBS) under the conditions of 37°C with 5% CO_2_. All cell culture reagents were purchased from Gibco Laboratories (NY, USA).

### Treatment with TCS

TCS was diluted with cell culture medium to concentrations from 0.25 to 32 μM. The cells were seeded into six-well flat-bottom plates (1 × 10^6^ cells/well) or 24-well glass-bottom plates (2 × 10^5^ cells/well) in serum-free and antibiotic-free DMEM medium.

### Transmission electron microscopy

For the transmission electron microscopy (TEM) observation, cells were incubated in six-well flat-bottom plates and were treated with TCS at a dose of 8 μM for 180 min. The cells were stained after immobilization. Autophagosome-like vesicles were observed under the TEM (Hitachi H-7650, Tokyo, Japan).

### Cell transfection

For the fluorescence microscope image collection, the cells needed to be transiently transfected with pEGFP-LC3 or ptfLC3 plasmids. The Raw264.7 cells were incubated in 24-well flat-bottom plates with glass slides. The instructions to complete cell transfection were followed. Then, we replaced the medium with new serum-free DMEM. The cells transfected with plasmids were used in other experiments.

### Confocal microscopy and immunofluorescence staining

Cells or transfected cells were seeded in 24-well glass-bottom plates. The cells were treated with TCS with or without *S*. typhimurium or *C. albicans*. The images were obtained by an Olympus FV1000 confocal laser-scanning microscope (Olympus, Tokyo, Japan) with a 60x objective lens. Image analyses and exports were performed using a Fluoview ver. 1.7.3.0 (Olympus, Tokyo, Japan).

### Western blotting

The cells were treated with TCS as described above, and the total protein was collected by centrifugation and was quantified using the BCA reagent (Beyotim, P0012). The images were obtained by a CanoScan LiDE 100 scanner (Canon). Protein blots were measured using Image-J software.

### Analysis of bacterial growth within MΦ

For the assay of the phagocytosis ratio in RAW264.7 cells, the cells were incubated in 24-well flat-bottom plates and were treated with Rapa (200 nM, 12 h), TCS, or TCS and 3-MA (5 mM, 180 min). The cells were then exposed to *S*. typhimurium or *C. albicans* at the respective MOI of 25:1 and 1:1 for 120 and 30 min. The assay of *S*. typhimurium growth in RAW264.7 cells was performed as previously described (Chiu et al., 2009). The collected *C. albicans* were spread onto YPD agar plates for CFU counting.

### Mice and *in vivo* studies

Female BALB/c mice (20 ± 2 g) were kept in conditions of a constant photoperiod (12 h light, 12 h dark) and had free access to food and water. For autophagy observation, mice were injected with TCS from 3.125 to 40 mg/kg for 90 min through the enterocoelia. Then, the mice were sacrificed for liver and spleen harvesting. Western blotting was used for the relevant protein assays.

For the assays assessing the effect of the bacteria-killing abilities, mice were infected by an intraperitoneal injection of an overnight culture of *S*. typhimurium (1 × 10^5^ organisms in 0.1 ml PBS). After 24 h, the mice were treated with vehicle (0.5% methylcellulose-0.1% Tween 80 in sterile water), TCS, or TCS and 3-MA (24 mg/kg) by intraperitoneal injection once a day for 4 days. Then, the mice were euthanized, and the livers and spleens were collected for homogenization in 5 ml cold PBS. The homogenates were then serially diluted and plated onto LB agar plates. The CFU counting was conducted as described above.

To establish a mouse model that was infected with *C. albicans*, mice were infected with *C. albicans* (1 × 10^7^ organisms in 0.1 ml PBS) by oral administration. After 3 days, the mice were randomly assigned to control and treatment groups. *C. albicans* were treated the same way as those treated with *S*. typhimurium. The homogenates were then serially diluted and plated onto YPD agar plates. The CFU counting was conducted as previously described.

For the long-term sub-inhibitory concentration animal administration experiment, mice were randomly assigned to control, experiment, and treatment groups. The mice were treated with vehicle, TCS (1, 2, or 3 mg/day), or TCS (3 mg/day) by oral ingestion of 15 ml and 20 μl of 3-MA (24 mg/kg) by intraperitoneal injection for 30 days.

### Intestinal bacteria cultivation and quantitation

Representative bacteria in the feces of the mice treated with vehicle, TCS (1, 2, or 3 mg/day), or TCS (3 mg/day) and 20 μl of 3-MA (24 mg/kg), were counted with differential media on the 30th day. Intestinal bacteria of mice were cultivated with differential media, including MRS medium, MacConkey agar, and Enterococcus faecalis agar, which were purchased from Hopebio Co., Ltd. (Qingdao, China). The MRS plate was incubated anaerobically for 2–3 days at 37°C, while the MacConkey and *E. faecalis* plates were incubated aerobically overnight at 37°C.

### 16S rRNA gene sequencing

16S rRNA gene high-throughput sequencing was used to determine the structure comparisons of the bacterial species in each of these samples using the Illumina HiSeq platform. PCR primers 515F and 806R targeting the bacterial 16S rRNA V4 region were selected for bacterial community analysis using the Illumina high-throughput sequencing method Sequences were analyzed using Quantitative Insights Into Microbial Ecology (QIIME) software and the UPARSE pipeline. The default settings for Illumina processing in QIIME were used and the UPARSE pipeline was then used to assign taxonomy at 97% similarity via the RDP classifier.

### Ethics statement

Mouse studies were performed in strict accordance with the Institutional Guiding Principles for Biomedical Research Involving Animals. The experiments were approved by the Jilin University Animal Care and Use Committee (no: IZ-2009-008). The gene copies indicated the absolute copy numbers present per unit of dry weight, whereas the normalized copy number based on 16S rRNA was regarded as the abundance. Sequences analysis were performed by Uparse software (Uparse v7.0.1001, http://drive5.com/uparse/). Sequences with ≥97% similarity were assigned to the same OTUs. The Silva Database (https://www.arb-silva.de/) was used based on RDP classifier (Version 2.2, http://sourceforge.net/projects/rdp-classifier/) algorithm to annotate taxonomic information.

### Statistical analysis

All of the results are expressed as the means ± *SD*. The group means were compared using a one-way ANOVA, and Student's *t*-test was used to determine the significance of differences. For *p* values,^*^p < 0.05, ^**^p < 0.01, or ^***^p < 0.001 compared with the control were considered statistically significant. The data are representative of triplicate experiments and are presented as the mean value ± the *SD*.

## Results

### TCS has inhibitory and killing activities against *S*. typhimurium or *C. albicans*

The MIC and MBC of TCS against the strain *S*. typhimurium ATCC14028 in this study were 0.5 μg/ml and 32 μM, respectively. The MIC and MBC of TCS against the strain *C. albicans* SC5314 tested in this study were 2 and 16 μg/ml, respectively, and this was consistent with a previous report that TCS against *C. albicans* is at 16 μg/ml (Higgins et al., 2012).

### TCS treatment induced an autophagic response in non-phagocytic cells (HeLa) or MΦ and *in vivo*

**Figure 1:**
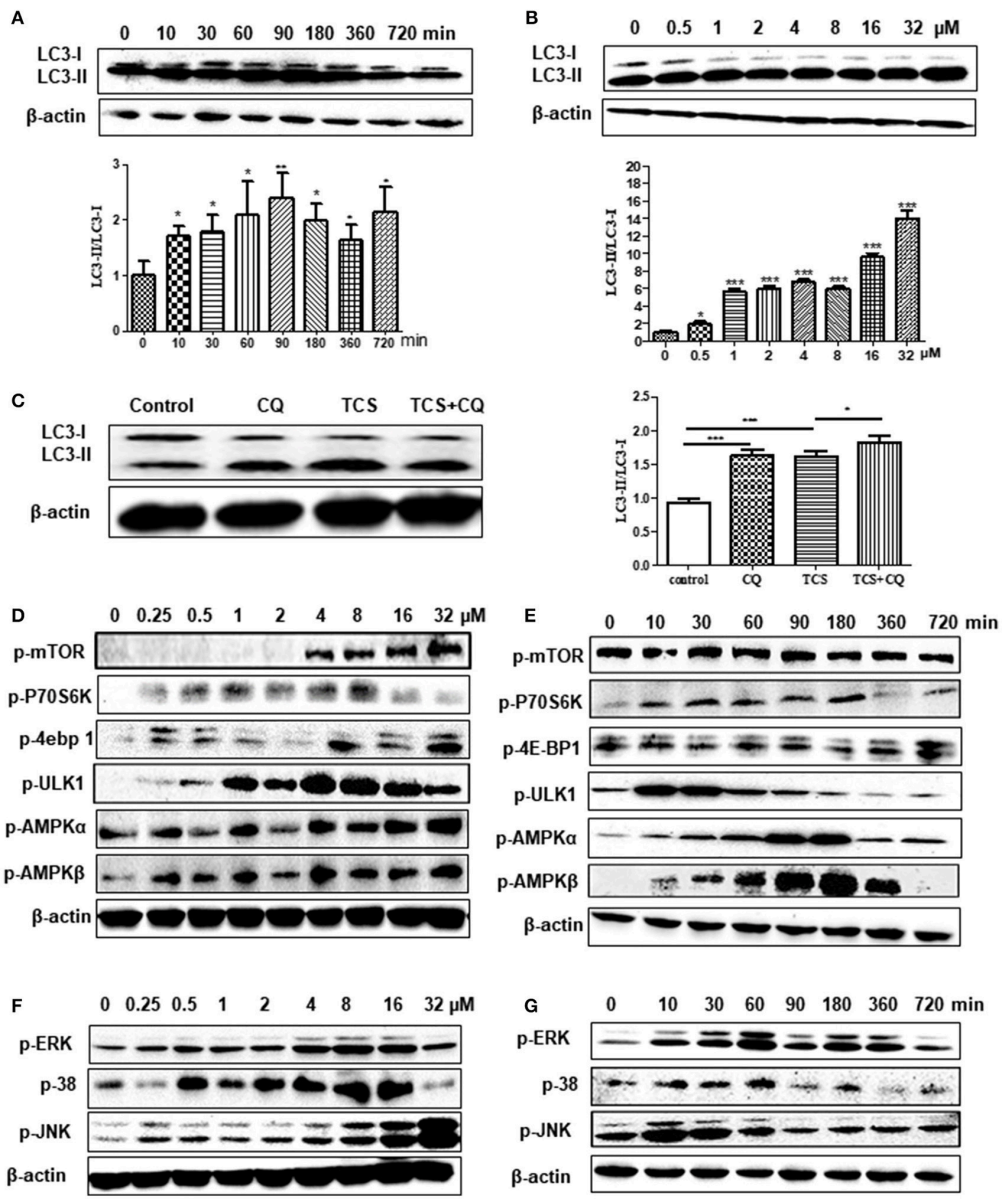
TCS treatment induces an autophagic response in MΦ. RAW264.7 cells were treated with TCS (8 μM) for different times ranging from 0 to 720 min. Western blotting was used to assay the expression of LC3 **(A)**. RAW264.7 cells were treated with TCS at different doses ranging from 0.5 to 32 μM for 90 min. Western blotting was used to assay the expression of LC3 **(B)**. RAW264.7 cells were pretreated with or without CQ (25 μM, 60 min) and were then treated with or without TCS (8 μM) for 90 min. The results was assayed by western blot **(C)**. RAW264.7 cells were treated with TCS at concentrations ranging from 0.25 to 32 μM for 90 min. The levels of phosphorylation of mTOR, p76S6K, 4E-BP1, AMPK-α, AMPK-β, and ULK1 **(D)** or JNK, ERK, and p38 **(F)** were assayed by western blot. RAW264.7 cells were treated with TCS (8 μM) for different times ranging from 0 to 720 min. The levels of phosphorylation of mTOR, p76S6K, 4E-BP1, AMPK-α, AMPK-β, and ULK1 **(E)** or JNK, ERK, and p38 **(G)** were assayed by western blot. Compared to the respective controls, ^*^p < 0.05, ^**^p < 0.01, ^***^p < 0.001.

To explore whether TCS induces autophagy in cells, TCS with different concentrations from 0.5 to 32 μM was added to HeLa cells that were transfected with GFP-LC3 plasmid. The results demonstrated that the number of LC3 puncta was significantly increased in the TCS- or Rapa-treated cells, whereas in the control and 3-MA-added cells, few LC3 puncta were found (Figure S1A). RAW264.7 cells were administered TCS for different times or treated with different doses for 90 min for western blot assays. The results demonstrated that the ratio of LC3-II/LC3-I increased, which was followed by a decrease and reached a peak after 90 min of treatment (Figure 1A). The ratio of LC3-II/LC3-I increased with the TCS dose (Figure 1B). To seek *in vivo* evidence that TCS induces autophagy, mice were treated with different doses of TCS, and the livers and spleens were collected for western blot assays. The results showed that TCS at all of the concentrations tested (3.75–25 mg/kg) improved the ratio of LC3-II/LC3-I both in the livers (Figure S1B) and spleens (Figure S1C) in a dose-dependent manner.

### TCS treatment increased autophagosome formation in MΦ

To further verify that autophagy was induced by TCS, we observed the autophagosome formation in TCS-treated RAW264.7 cells by TEM. In the control groups (Figure S2A) or 3-MA-treated groups (Figure S2B), no autophagosome was found. In the TCS-treated cells, autophagosomes with a double membrane structure could be easily found, and the TEM captured the moment that the autophagosome was fusing with a lysosome (Figure S2C). The same structures were seen in the rapamycin-treated groups (Figure S2D).

### Further verification of the autophagic response induced by TCS

To further reveal whether TCS induced an autophagic response, firstly, non-phagocytic cells (HeLa) were transfected with ptf-LC3 plasmids and were treated with TCS. The GFP-LC3 component is sensitive to lysosomal proteolysis, and therefore, the corresponding GFP fluorescence is quenched once it undergoes proteolysis, whereas the RFP-LC3 component will not be quenched with proteolysis. The results showed that compared with the control groups, the numbers of both the GFP-LC3 and RFP-LC3 puncta in TCS-treated cells increased, and in the merged images, red and yellow puncta were found (Figure S2E). This demonstrated that during this autophagic process, the fluorescence of some GFP-LC3 quenched, and thus, the autophagosome and lysosome fused in TCS-induced autophagy. Secondly, we use chloroquine (CQ) to verify that TCS induced autophagy. The results of western blot assays showed that in TCS- or CQ-treated RAW264.7 cells, the ratio of LC3-II/LC3-I increased compared with the TCS-alone-treated cells, and the ratio of LC3-II/LC3-I in the cells treated with TCS and CQ was significantly increased (Figure 1C). This demonstrated that during the process of TCS-induced autophagy, the addition of CQ prevented the fusion of the autophagosome and lysosome and resulted in the accumulation of LC3-II. Thus, the procedure of the autophagosome fused with a lysosome was included in TCS-induced autophagy. Overall, these results further suggest that TCS treatment induces an autophagic response.

### TCS stimulated autophagy through the AMPK/ULK1 pathway instead of the mTOR-dependent pathway in MΦ

To explore whether TCS-induced autophagy via the classical mTOR-dependent pathway, RAW264.7 cells were treated with TCS at different concentrations from 0.25 to 32 μM for 90 min. Western blot assays showed that with the increased concentration, the phosphorylation of mTOR, p70S6K, and 4E-BP1 increased compared to the untreated cells (Figure 1D). After treatment with TCS (8 μM), the western blot results showed that during the treatment period (0–720 min), the phosphorylation of mTOR, p70S6K, or 4E-BP1 was not downregulated (Figure 1E). Furthermore, the cells were treated with TCS and Torin1 (an mTOR inhibitor), and the results showed that Torin1 had no effect on the expression of LC3-II (Figure S3A). Therefore, TCS triggered mTOR-independent autophagy in MΦ.

Furthermore, the western blot assay showed that TCS up-regulated the phosphorylation of AMPK and ULK1 in RAW264.7 cells treated with TCS at concentrations from 0.25 to 32 μM for 90 min (Figure 1D). After the treatment with TCS (8 μM) for the different times, ranging from 0 to 720 min, the western blot results showed that with increased treatment time, the phosphorylation of AMPK and ULK1 trended to rise at first and then fall (Figure 1E). In addition, the cells were treated with TCS and compound C (an AMPK inhibitor) to better understand this pathway. The results showed that compound C decreased AMPK and ULK1 phosphorylation and decreased the level of LC3-II expression induced by TCS (Figure S3B). Therefore, TCS stimulated autophagy through AMPK/ULK1 pathway activation.

### TCS stimulated autophagy through JNK/p38/ERK pathway activation

Next, the cells were treated with TCS at concentrations from 0.25 to 32 μM for 90 min. The western blot assay demonstrated that the levels of phosphorylation of JNK, p38, and ERK were up-regulated in a dose-dependent manner in general (Figure 1F). After treatment with TCS (8 μM) for different times, ranging from 0 to 720 min, the western blot results indicated that phosphorylation of JNK, p38, and ERK presented a trend that increased at first and then fell with time (Figure 1G).

To explore the regulatory relationship among JNK, p38, and ERK in TCS-induced autophagy, the corresponding inhibitors SP600126, SB203580, and PD98059 for JNK, p38, and ERK were used. The results showed that when PD98059 was added, the expression of p-ERK was inhibited, whereas the expression of p-JNK and p-p38 was not affected (Figure S3C). When SB203580 was added, the phosphorylation of p38 and ERK was inhibited, but it had no influence on the phosphorylation of JNK (Figure S3C). SP600126 was also used to pretreat the cells, and the phosphorylation levels of JNK, p38 and ERK were all inhibited (Figure S3D). Meanwhile, the ratio of LC3-II/LC3-I was reduced when the three types of inhibitors were added (Figures S3C,D). These results demonstrated that TCS stimulated autophagy through JNK/p38/ERK pathway activation. Moreover, JNK was upstream of p38 and ERK and regulated the levels of their phosphorylation. As the upstream regulator of ERK, p38 also played a regulatory role in the phosphorylation of ERK.

### TCS improved the p62 or NDP52 expression and strengthened the co-localization among intracellular pathogens, LC3 and p62, or NDP52

**Figure 2:**
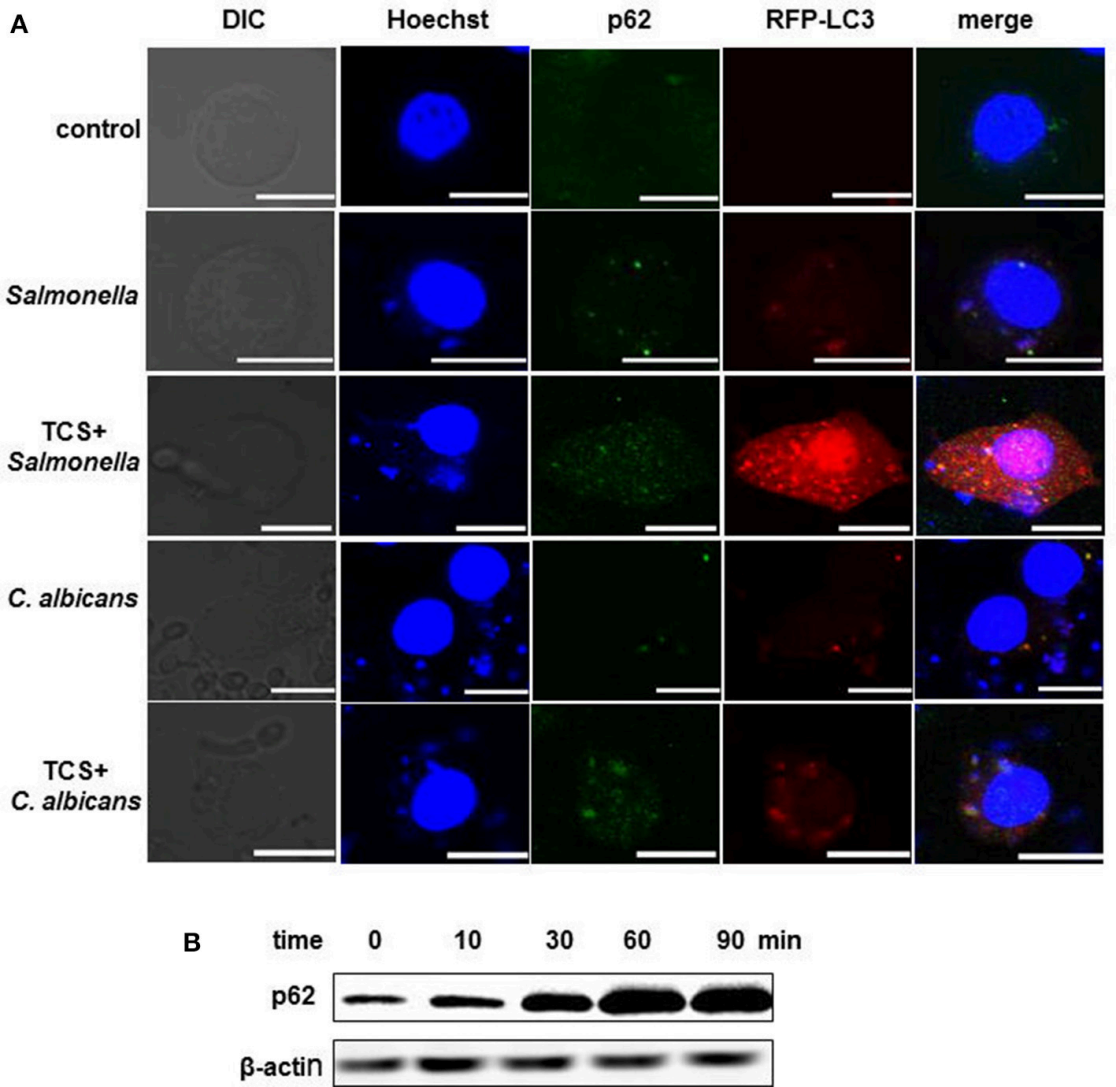
TCS improves p62 expression and strengthens the co-localization among intracellular pathogens, LC3 and p62. RAW264.7 cells were transfected with RFP-LC3 plasmids and incubated with or without pathogens for 30 min, and then the cells were treated with or without TCS (8 μM) for 90 min, followed by immunofluorescent staining. p62 was marked by a p62 antibody, and the pathogens and nuclei were stained by Hoechst 33342. The results were collected by a laser-scanning confocal microscope **(A)**. Scale bars = 10 μm. The cells were treated with TCS (8 μM) for different times ranging from 0 to 90 min, and the levels of p62 were assayed by western blot **(B)**. Compared to the respective controls.

RAW264.7 cells were transfected with RFP-LC3 plasmids and were incubated with or without pathogens. The cells infected with pathogens were then treated with or without TCS. Then, the images were collected by a confocal laser-scanning microscope. The results showed that in the controls, few p62 or NDP52 and LC3 puncta could be found. There was a co-localization among intracellular pathogens, LC3 and p62, or NDP52 in the *S*. typhimurium or *C. albicans* infected cells, and these co-localizations were strengthened by TCS treatment (Figure 2A, Figure S4). After treatment with TCS (8 μM) at different times, ranging from 0 to 90 min, the western blot results showed that with the increase in treatment time, the expression of p62 increased (Figure 2B). Together, the results confirmed that TCS improved the p62 and NDP52 expression and strengthened the co-localization among intracellular pathogens, NDP52 or p62 and LC3.

### The ability of pathogen clearance in RAW264.7 cells was enhanced by TCS treatment

**Figure 3:**
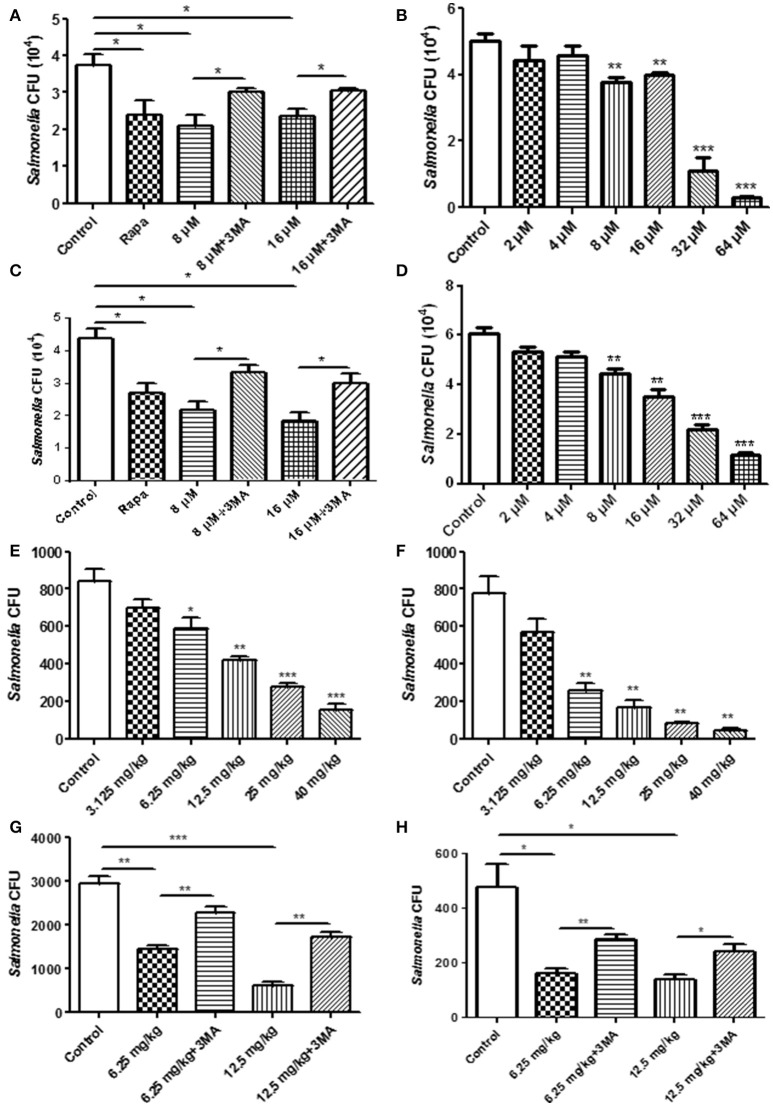
The pathogen clearance ability is enhanced by TCS treatment *in vitro* and *in vivo*. RAW264.7 cells were incubated with Rapa (200 nM, 12 h), 3-MA (5 mM, 180 min), and TCS (8 μM, 90 min) **(A)** or TCS (0–64 μM, 90 min) **(B)** first and then infected with *S*. typhimurium at an MOI of 25:1. Colony counting was used to calculate the extracellular CFU. RAW264.7 cells were incubated with *S*. typhimurium at an MOI of 25:1 for 120 min. The cells were then treated with Rapa (200 nM, 12 h), 3-MA (5 mM, 180 min), and TCS (8 μM, 90 min) **(C)** or TCS (0–64 μM, 90 min) **(D)** for 90 min. Colony counting was used to calculate the intracellular CFU. Female BALB/c mice were infected with *S*. typhimurium for 24 h, and the mice were treated with TCS once a day for 4 days. The livers **(E)** and spleens **(F)** were homogenized for colony counting. BALB/c mice were infected with *S*. typhimurium for 24 h, and the mice were treated with or without 3-MA (24 mg/kg) first and were then injected with TCS once a day for 4 days. The livers and spleens were homogenized for colony counting **(G,H)**. Compared to the respective controls, ^*^p < 0.05, ^**^p < 0.01, ^***^p < 0.001.

For the phagocytosis rate assays, RAW264.7 cells were treated with TCS first and were then incubated with *S*. typhimurium or *C. albicans*. The number of extracellular pathogens was obtained by colony counting. The results demonstrated that the number of extracellular pathogens decreased when the cells were treated with TCS or Rapa, and the decrease was prevented by 3-MA treatment (Figure 3A, Figure S5A). In addition, for the higher doses of TCS, there was a lower number of extracellular pathogens (Figure 3B, Figure S5B). For the pathogen killing assays, RAW264.7 cells were incubated with *S*. typhimurium or *C. albicans* and were then treated with TCS. The results showed that the number of intracellular pathogens decreased with the increasing dose of TCS treatment (Figure 3C, Figure S5C), and the decrease in colony number was prevented by 3-MA treatment (Figure 3D, Figure S5D). Taken together, we concluded that the phagocytic and killing capacity of pathogens by RAW264.7 cells was enhanced by TCS treatment in a dose-dependent manner.

### The ability of pathogen clearance was enhanced by TCS treatment *in vivo*

To seek *in vivo* evidence that TCS strengthens the killing of pathogens by inducing autophagy, we established a mouse model infected with *S*. typhimurium. The livers and spleens were harvested for bacterial colony counting after 4 days of TCS therapy. With higher doses of TCS, there were fewer surviving *S*. typhimurium in the livers (Figure 3E) and spleens (Figure 3F), whereas the 3-MA treatment prevented the *S*. typhimurium from being killed by TCS-induced autophagy both in the livers and spleens (Figures 3G,H). In addition, the mice were infected with *C. albicans* by oral administration, and the therapy by different concentrations of TCS was conducted three times a day. The tongues were collected for colony counting. The results demonstrated that TCS at concentrations from 6.25 to 20 mg/kg decreased the number of surviving *C. albicans* in the tongues in a dose-dependent manner (Figure S5E). 3-MA treatment inhibited the TCS-induced autophagy and protected the *C. albicans* from being killed (Figure S5F). Together, our results demonstrated that TCS strengthens the killing of pathogens by inducing autophagy *in vivo*.

### The intestinal flora balance was broken by long-term TCS administration in mice

**Figure 4:**
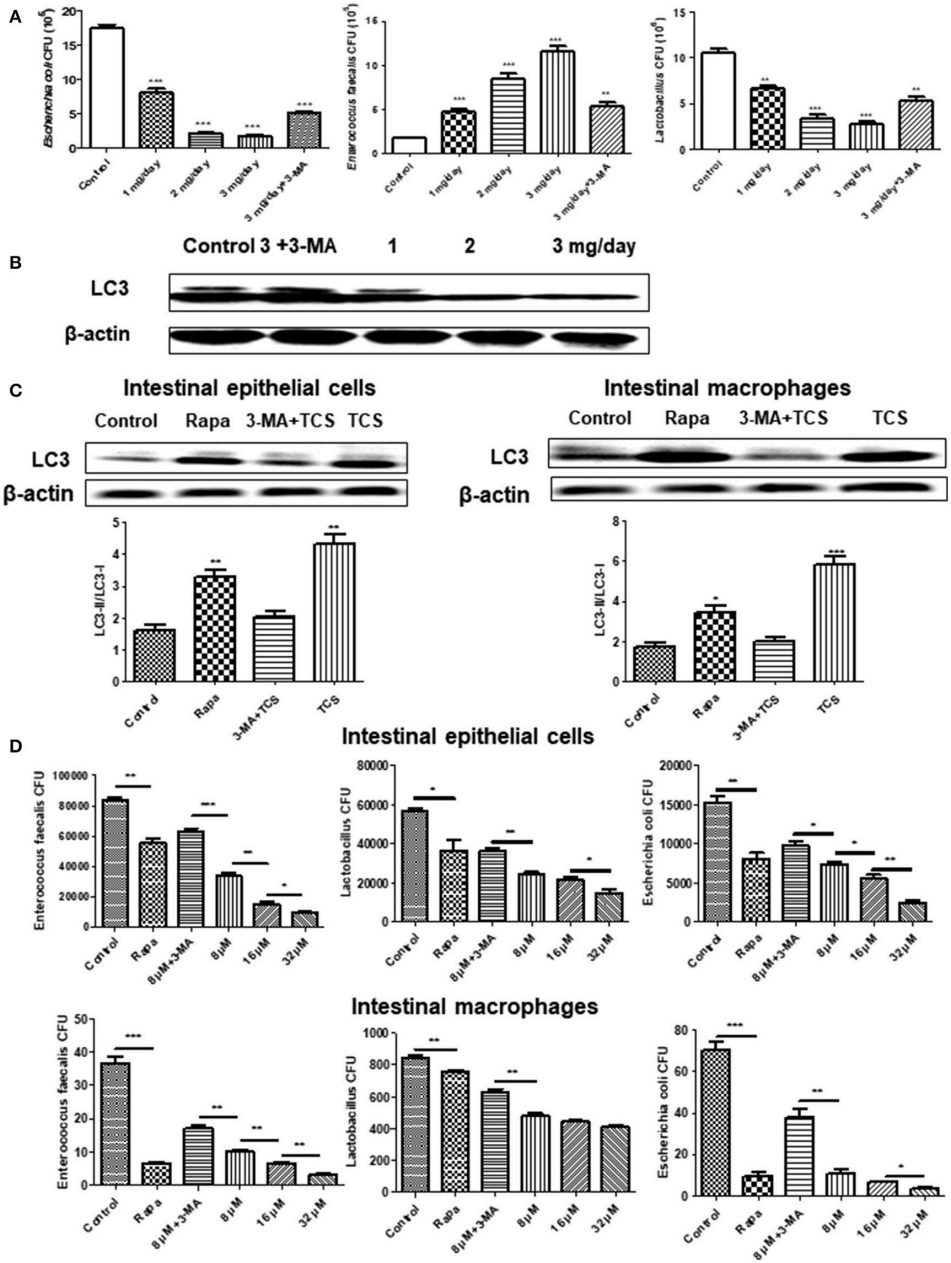
The effects of long-term TCS administration on intestinal microbes in mice. Female BALB/c mice were treated with vehicle (0.5% methylcellulose−0.1% Tween 80 in sterile water), TCS (1, 2, 3 mg/day), or TCS (3 mg/day) by oral ingestion of 15 ml and with 20 μl of 3-MA (24 mg/kg) by an intraperitoneal injection for 30 days. Colony counting was used to calculate the major intestinal flora (*E. faecalis, Lactobacillus*, and *E. coli*) CFU **(A)**. A western blot was used to assay the expressions of LC3 in the mouse intestine **(B)**. A western blot was used to assay the expressions of LC3 in the extracted mouse intestinal epithelial cells and macrophages incubated with Rapa (200 nM, 12 h), TCS (8 μM, 90 min), or 3-MA (5 mM, 180 min) **(C)**. The extracted mouse intestinal epithelial cells and macrophages were incubated with Rapa (200 nM, 12 h), TCS (8 μM, 90 min), or 3-MA (5 mM, 180 min) first and were then infected with the major intestinal flora (*E. faecalis, Lactobacillus*, and *E. coli*) at an MOI of 25: 1. Colony counting was used to calculate the intracellular CFU **(D)**. Compared to the respective controls, ^*^p < 0.05, ^**^p < 0.01, ^***^p < 0.001.

To explore the effect of TCS on autophagy in the intestine and intestinal flora *in vivo*, we established a mouse model of a long-term sub-inhibitory concentration of TCS administration. The major intestinal flora in the feces of the mouse model were counted, and the total number of intestinal flora was significantly decreased with the increased TCS concentration. Moreover, the number of *Lactobacillus* and *E. coli* significantly decreased, but the *E. faecalis* significantly increased (Figure 4A). The shifts of the bacterial community compositions were further corroborated by clear clustering of the dominant bacterial genus corresponding to different treatments in the heat map as shown in Figure S6. Firmicutes and Bacteroidetes had higher relative abundances compared with other phyla. Lactobacillus and Escherichia-Shigella had the similar results with Figure 4A. In addition, the western blot results of LC3 confirmed that autophagy occurred in the intestine of the mouse model and that autophagy was more significant with the increased TCS concentration (Figure 4B). To verify the above results, mouse intestinal macrophages and non-phagocytic cells (epithelial cells) were extracted and treated with TCS to induce autophagy. The results showed that the ratio of LC3-II/LC3-I under treatment of TCS or Rapa increased compared to the treatment of the combination of TCS/3-MA or control in both the mouse intestinal macrophages and epithelial cells (Figure 4C). Furthermore, the intracellular pathogen clearance experiments showed that TCS, in a dose-dependent manner, enhanced the killing of the strains *Lactobacillus, E. coli*, and *E. faecalis* isolated from mouse intestine by extracting the intestinal macrophages and epithelial cells, whereas 3-MA treatment reversed the results, suggesting that TCS-induced autophagy played a role in enhancing intracellular pathogen clearance (Figure 4D). Taken together, the results showed that the intestinal flora balance of the mouse was broken by long-term sub-inhibitory concentrations of TCS treatment.

## Discussion

The mouse *S*. typhimurium infection model is significant for the study of murine and human typhoid diseases. In this study, our results showed that different concentrations of TCS contributed to the reduction of the number of *S*. typhimurium in the liver and spleen, and this effect was reversed by 3-MA. *C. albicans* is a classic opportunistic pathogen, and the clinical application of antibiotics and immunosuppressive agents leads to the occurrence of a *C. albicans* disease increase (Shin et al., 2013), whereas our results showed that TCS enhanced the killing of *C. albicans* in a mouse oral *C. albicans* infection model, and this effect was reversed by 3-MA. All of these results suggested that TCS induced autophagy to enhance the microbiocidal effect in mammalian cells *in vivo*.

Our TEM results showed that TCS treatment caused the autophagosome to fuse to lysosomes, which was presented as the quenching of GFP fluorescence in the merged image of TCS-treated non-phagocytic cells (HeLa). Furthermore, our western blot results further verified that TCS induced autophagy. The occurrence of autophagy is a complex and conservative process in which mTOR is a negative regulator. However, in this study, the western blot results confirmed that TCS treatment inhibited mTOR activity and the phosphorylation of 70S6K and 4E-BP1, and the pharmacological inhibition assays also indicated that TCS did not regulate the occurrence of autophagy through the mTOR pathway.

ULK1 is the initiation molecule of autophagy. AMPK is a positive regulator of ULK1, which activates the phosphorylation of ULK1 to initiate autophagy. In this study, our results showed that different concentrations of TCS induced the phosphorylation of AMPK and ULK1, and the induced intensity of AMPK and ULK1 increased with time. The results suggested that TCS activates the AMPK/ULK1 pathway to induce autophagy. ERK, p38, and JNK are involved in the induction of autophagy, but these three kinases play different roles in different cell types with different inducing factors (Bhui et al., 2010). In this study, our results suggested that TCS induced autophagy by activating the ERK, p38, and JNK pathways of the MAPK family, and specifically, we identified that JNK is the upstream regulatory factor of p38 and that p38 is the upstream regulatory factor of ERK in TCS-induced autophagy of RAW264.7 cells.

Ubiquitin is a small molecule in eukaryotic cells that tags the protein or pathogen that needs to be degraded by the various ubiquitin-catalyzed enzymes. NDP52 and p62 are ubiquitin receptor proteins that were discovered recently. In this study, the results showed that TCS treatment significantly increased the expression of NDP52, p62, and LC3 and their co-localization with pathogens. These results proved that TCS strengthened the expression of ubiquitin receptor proteins in macrophages to enhance the identification of pathogens.

In this study, we established a mouse model of a long-term sub-inhibitory concentration of TCS administration, and the results showed that TCS disrupted the intestinal flora balance. Gaulke et al. also showed that TCS altered the composition and ecological dynamics of microbial communities in the gut in adult zebrafish (Gaulke et al., 2016). However, Poole et al. showed that routine personal care use with TCS neither exerts a major influence on the microbial communities in the gut and mouth nor alters markers of endocrine function in humans, but the authors believed that their study was limited by the small sample size and the imprecise administration of household and personal care products (HPCPs) (Poole et al., 2016). Our study for the first time demonstrated that TCS disturbed the balance of the intestinal flora by autophagy.

In this study, our results of *in vitro* intestinal epithelial cells and macrophages showed that TCS killed *E. faecalis, Lactobacillus*, and *E. coli* by inducing autophagy in the two types of cells. Moreover, the *in vivo* results showed that the number of *Lactobacillus* and *E. coli* decreased, and surprisingly, the number of *E. faecalis* increased and the shifts of the bacterial community compositions were further corroborated by 16S rRNA gene sequencing. Previous research clarified that *Lactobacillus* and *E. coli* are killed by TCS (McMurry et al., 1998; Wicht et al., 2005), but *E. faecalis* has a relatively high-level intrinsic TCS resistance (Zhu et al., 2013). *Lactobacillus, E. coli*, and *E. faecalis* colonize in different regions in the intestine (Guarner and Malagelada, 2003). We speculated that because *E. faecalis* is mainly distributed in the rectum, where the TCS content is relatively lower compared with other parts of the intestine (Delmas et al., 2007), the killing effect of *E. faecalis* by TCS-induced autophagy may be lower in the rectum. Previous studies demonstrated that a sub-inhibitory concentration of antibiotics leads to the increased expression of bacterial resistance genes and causes their horizontal transmission (Davies et al., 2006; Palmer et al., 2010). In addition, Gieffers et al. also demonstrated that sub-inhibitory concentrations of antibiotics increased the number of pathogens in visceral organs (Gieffers et al., 2004), which was similar to our results demonstrating that *E. faecalis* was increased by TCS treatment *in vivo*. Taken together, our study confirmed that a sub-inhibitory concentration of TCS-induced autophagy broke the balance of the intestinal microflora in mice.

A previous study showed that autophagy acts as a double-edged sword in a variety of diseases (Shintani and Klionsky, 2004). There is evidence that autophagy is oncogenic in some contexts (Perlmutter, 2009; Rosenfeldt and Ryan, 2011) and that p62 is required for tumorigenesis *in vitro* and *in vivo* (Duran et al., 2008). Moreover, some studies revealed that mTOR mechanisms are responsible for hepatocarcinogenesis, and activating the mTOR pathway promotes tumorigenesis in multiple organs (Memmott and Dennis, 2010; Ho et al., 2012). In this study, our results confirmed that TCS induced autophagy in the mouse intestine, liver, and spleen. Furthermore, our western blot and immunofluorescence results showed that TCS increased the expression of p62 in RAW264.7 cells. In addition, our results also showed that the phosphorylation of mTOR, 70S6K, and 4E-BP1 increased under the treatment of TCS. Therefore, we inferred that TCS has a carcinogenic tendency by inducing autophagy. Yueh et al. also indicated that TCS is capable of stimulating liver cell proliferation and fibrotic responses, strongly enhances hepatocarcinogenesis and accelerates hepatocellular carcinoma development in mice (Yueh et al., 2014).

Overall, we demonstrated that TCS induced autophagy in macrophages and non-phagocytic cells *in vivo* and *in vitro*. In addition, sub-lethal concentrations of TCS enhanced the clearing of *S*. typhimurium or *C. albicans* in infected MΦ and *in vivo*. Noteworthy, TCS broke the balance of the intestinal microflora and has a carcinogenic tendency.

## Author contributions

LY, CW, and XS participated in the design of this study, and they performed the statistical analysis. LY carried out the study and collected important background information. CW and XS drafted the manuscript. All authors read and approved the final manuscript. CW, XS, YW, and YA carried out the concepts, design, definition of intellectual content, literature search, data analysis and manuscript preparation. ZY completed the additional experiments requested by the reviewers. SL, YL, XfW, and WL provided assistance for data acquisition, data analysis and statistical analysis. XT and XyW carried out literature search, data acquisition and manuscript editing. ZC and ML performed manuscript review. All authors have read and approved the content of the manuscript.

### Conflict of interest statement

The authors declare that the research was conducted in the absence of any commercial or financial relationships that could be construed as a potential conflict of interest.

## References

[B1] Bhui K. Tyagi S. Prakash B. Shukla Y. (2010). Pineapple bromelain induces autophagy, facilitating apoptotic response in mammary carcinoma cells. Biofactors 36, 474–482. 10.1002/biof.121 20848558

[B2] Cemma M. Kim P. K. Brumell J. H. (2011). The ubiquitin-binding adaptor proteins p62/SQSTM1 and NDP52 are recruited independently to bacteria-associated microdomains to target Salmonella to the autophagy pathway. Autophagy 7, 341–345. 10.4161/auto.7.3.14046 21079414 PMC3060414

[B3] Chiu H. C. Kulp S. K. Soni S. Wang D. Gunn J. S. Schlesinger L. S.. (2009). Eradication of intracellular *Salmonella enterica* serovar Typhimurium with a small-molecule, host cell-directed agent. Antimicrob. Agents Chemother. 53, 5236–5244. 10.1128/AAC.00555-09 19805568 PMC2786354

[B4] Clemente J. C. Ursell L. K. Parfrey L. W. Knight R. (2012). The impact of the gut microbiota on human health: an integrative view. Cell 148, 1258–1270. 10.1016/j.cell.2012.01.035 22424233 PMC5050011

[B5] Davies J. Spiegelman G. B. Yim G. (2006). The world of subinhibitory antibiotic concentrations. Curr. Opin. Microbiol. 9, 445–453. 10.1016/j.mib.2006.08.006 16942902

[B6] Delmas J. Robin F. Schweitzer C. Lesens O. Bonnet R. (2007). Evaluation of a new chromogenic medium, ChromID VRE, for detection of vancomycin-resistant Enterococci in stool samples and rectal swabs. J. Clin. Microbiol. 45, 2731–2733. 10.1128/JCM.00448-07 17553971 PMC1951253

[B7] Duran A. Linares J. F. Galvez A. S. Wikenheiser K. Flores J. M. Diaz-Meco M. T.. (2008). The signaling adaptor p62 is an important NF-kappaB mediator in tumorigenesis. Cancer Cell 13, 343–354. 10.1016/j.ccr.2008.02.001 18394557

[B8] Fang J. L. Stingley R. L. Beland F. A. Harrouk W. Lumpkins D. L. Howard P. (2010). Occurrence, efficacy, metabolism, and toxicity of triclosan. J. Environ. Sci. Health C Environ. Carcinog. Ecotoxicol. Rev. 28, 147–171. 10.1080/10590501.2010.504978 20859822

[B9] Gaulke C. A. Barton C. L. Proffitt S. Tanguay R. L. Sharpton T. J. (2016). Triclosan exposure is associated with rapid restructuring of the microbiome in adult zebrafish. PLoS ONE 11:e0154632. 10.1371/journal.pone.0154632 27191725 PMC4871530

[B10] Gieffers J. Rupp J. Gebert A. Solbach W. Klinger M. (2004). First-choice antibiotics at subinhibitory concentrations induce persistence of *Chlamydia pneumoniae*. Antimicrob. Agents Chemother. 48, 1402–1405. 10.1128/AAC.48.4.1402-1405.2004 15047553 PMC375316

[B11] Guarner F. Malagelada J. R. (2003). Gut flora in health and disease. Lancet 361, 512–519. 10.1016/S0140-6736(03)12489-0 12583961

[B12] Higgins J. Pinjon E. Oltean H. N. White T. C. Kelly S. L. Martel C. M.. (2012). Triclosan antagonizes fluconazole activity against *Candida albicans*. J. Dent. Res. 91, 65–70. 10.1177/0022034511425046 21972257 PMC3232117

[B13] Ho C. Wang C. Mattu S. Destefanis G. Ladu S. Delogu S.. (2012). AKT (v-akt murine thymoma viral oncogene homolog 1) and N-Ras (neuroblastoma ras viral oncogene homolog) coactivation in the mouse liver promotes rapid carcinogenesis by way of mTOR (mammalian target of rapamycin complex 1), FOXM1 (forkhead box M1)/SKP2, and c-Myc pathways. Hepatology 55, 833–845. 10.1002/hep.24736 21993994 PMC3269553

[B14] Kimura S. Noda T. Yoshimori T. (2007). Dissection of the autophagosome maturation process by a novel reporter protein, tandem fluorescent-tagged LC3. Autophagy 3, 452–460. 10.4161/auto.4451 17534139

[B15] Macartney K. K. Baumgart D. C. Carding S. R. Brubaker J. O. Offit P. A. (2000). Primary murine small intestinal epithelial cells, maintained in long-term culture, are susceptible to rotavirus infection. J. Virol. 74, 5597–5603. 10.1128/JVI.74.12.5597-5603.2000 10823867 PMC112047

[B16] McMurry L. M. Oethinger M. Levy S. B. (1998). Triclosan targets lipid synthesis. Nature 394, 531–532. 10.1038/28970 9707111

[B17] Memmott R. M. Dennis P. A. (2010). The role of the Akt/mTOR pathway in tobacco carcinogen-induced lung tumorigenesis. Clin. Cancer Res. 16, 4–10. 10.1158/1078-0432.CCR-09-0234 20028747 PMC2805044

[B18] Moore L. E. Ledder R. G. Gilbert P. McBain A. J. (2008). *In vitro* study of the effect of cationic biocides on bacterial population dynamics and susceptibility. Appl. Environ. Microbiol. 74, 4825–4834. 10.1128/AEM.00573-08 18515475 PMC2519354

[B19] Nicola A. M. Albuquerque P. Martinez L. R. Dal-Rosso R. A. Saylor C. De Jesus M.. (2012). Macrophage autophagy in immunity to *Cryptococcus neoformans* and *Candida albicans*. Infect. Immun. 80, 3065–3076. 10.1128/IAI.00358-12 22710871 PMC3418760

[B20] Palmer K. L. Kos V. N. Gilmore M. S. (2010). Horizontal gene transfer and the genomics of enterococcal antibiotic resistance. Curr. Opin. Microbiol. 13, 632–639. 10.1016/j.mib.2010.08.004 20837397 PMC2955785

[B21] Perlmutter D. H. (2009). Autophagic disposal of the aggregation-prone protein that causes liver inflammation and carcinogenesis in alpha-1-antitrypsin deficiency. Cell Death Differ. 16, 39–45. 10.1038/cdd.2008.103 18617899

[B22] Poole A. C. Pischel L. Ley C. Suh G. Goodrich J. K. Haggerty T. D.. (2016). Crossover control study of the effect of personal care products containing triclosan on the microbiome. mSphere 1:e00056–15. 10.1128/mSphere.00056-15 27303746 PMC4888890

[B23] Rosenfeldt M. T. Ryan K. M. (2011). The multiple roles of autophagy in cancer. Carcinogenesis 32, 955–963. 10.1093/carcin/bgr031 21317301 PMC3128556

[B24] Shin S. H. Lee Y. S. Shin Y. P. Kim B. Kim M. H. Chang H. R.. (2013). Therapeutic efficacy of halocidin-derived peptide HG1 in a mouse model of *Candida albicans* oral infection. J. Antimicrob. Chemother. 68, 1152–1160. 10.1093/jac/dks513 23302580

[B25] Shintani T. Klionsky D. J. (2004). Autophagy in health and disease: a double-edged sword. Science 306, 990–995. 10.1126/science.1099993 15528435 PMC1705980

[B26] Sun S. Li Y. Guo Q. Shi C. Yu J. Ma L. (2008). *In vitro* interactions between tacrolimus and azoles against *Candida albicans* determined by different methods. Antimicrob. Agents Chemother. 52, 409–417. 10.1128/AAC.01070-07 18056277 PMC2224779

[B27] U.S. FDA (2016). FDA issues final rule on safety and effectiveness of antibacterial soaps. FDA News Release. Available online at: https://www.fda.gov/NewsEvents/Newsroom/PressAnnouncements/ucm517478.htm

[B28] Wang J. Kang R. Huang H. Xi X. Wang B. Wang J.. (2014). Hepatitis C virus core protein activates autophagy through EIF2AK3 and ATF6 UPR pathway-mediated MAP1LC3B and ATG12 expression. Autophagy 10, 766–784. 10.4161/auto.27954 24589849 PMC5119055

[B29] Wicht M. J. Haak R. Kneist S. Noack M. J. (2005). A triclosan-containing compomer reduces Lactobacillus spp. predominant in advanced carious lesions. Dent. Mater. 21, 831–836. 10.1016/j.dental.2004.09.011 15876453

[B30] Wu Y. Chitranshi P. Loukotková L. Gamboa da Costa G. Beland F. A. Zhang J.. (2017). Cytochrome P450-mediated metabolism of triclosan attenuates its cytotoxicity in hepatic cells. Arch. Toxicol. 91, 2405–2423. 10.1007/s00204-016-1893-6 27896399

[B31] Yu L. Ling G. Deng X. Jin J. Jin Q. Guo N. (2011). *In vitro* interaction between fluconazole and triclosan against clinical isolates of fluconazole-resistant *Candida albicans* determined by different methods. Antimicrob. Agents Chemother. 55, 3609–3612. 10.1128/AAC.01313-10 21576450 PMC3122452

[B32] Yueh M. F. Taniguchi K. Chen S. Evans R. M. Hammock B. D. Karin M.. (2014). The commonly used antimicrobial additive triclosan is a liver tumor promoter. Proc. Natl. Acad. Sci. U.S.A. 111, 17200–17205. 10.1073/pnas.1419119111 25404284 PMC4260592

[B33] Zhu L. Bi H. Ma J. Hu Z. Zhang W. Cronan J. E. (2013). The two functional enoyl-acyl carrier protein reductases of *Enterococcus faecalis* do not mediate triclosan resistance. MBio 4:e00613–13. 10.1128/mBio.00613-13 PMC3791895 24085780

