# Supplementary material for: Triclosan Enhances the Clearing of Pathogenic Intracellular Salmonella or Candida albicans but Disturbs the Intestinal Microbiota through mTOR-Independent Autophagy
Source: Front Cell Infect Microbiol. 2018 Feb 21;8:49. doi: 10.3389/fcimb.2018.00049 (PMC5826388; doi:10.3389/fcimb.2018.00049)
Supplement: Supplementary file 1 [file Image1.PDF]

Fig. S1 TCS treatment induced autophagic response in nonphagocytic cells (HeLa) and *in vivo.*

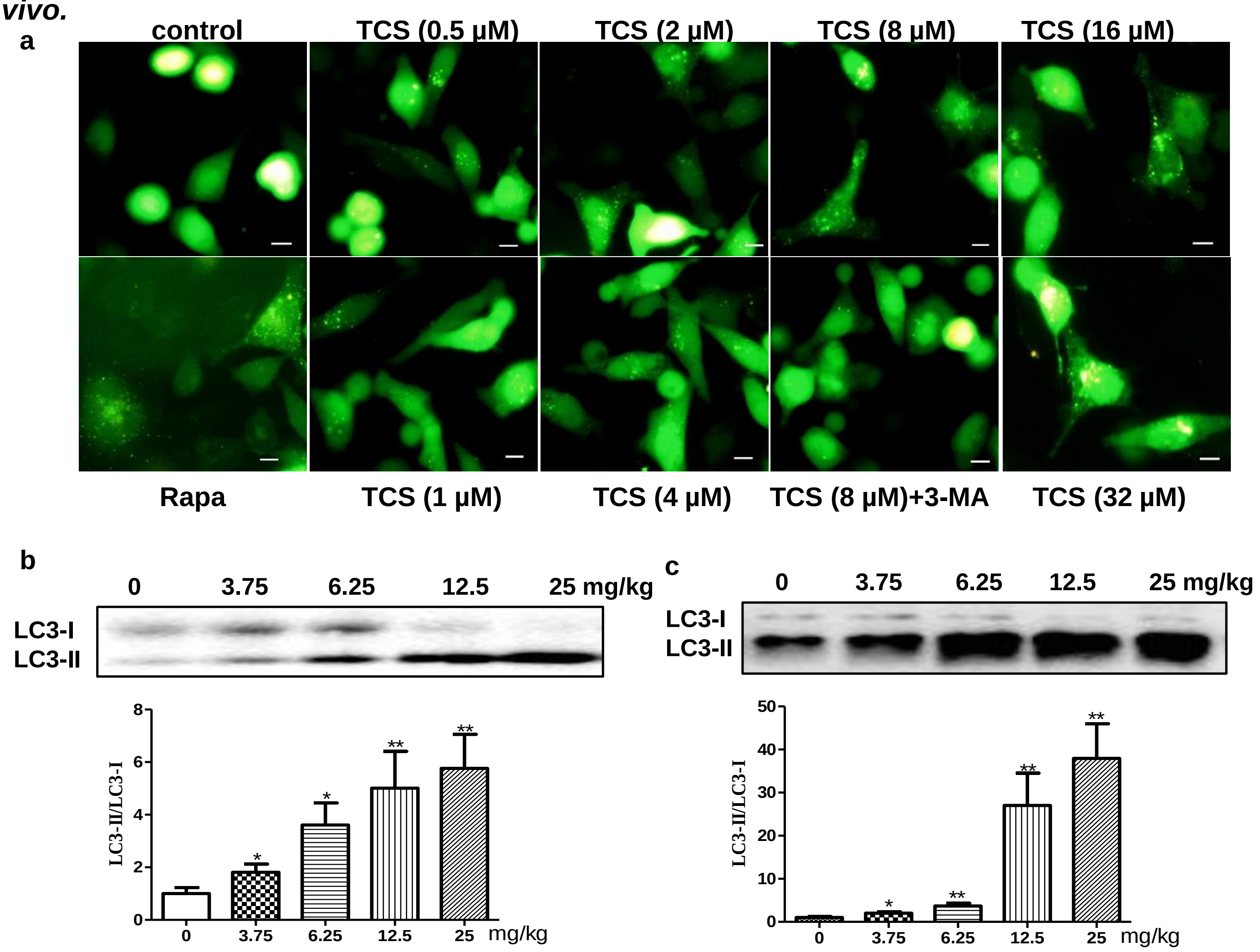

Fig. S1 TCS treatment induced autophagic response in nonphagocytic cells (HeLa) and *in vivo.* The HeLa cells were trasfected with GFP-LC3 plasmids for 12 h and treated with TCS with different doses from 0.5 to 32 μM, Rapa (200 nM) or TCS (8 μM) and 3-MA (5 mM), the groups added DMSO were used as control, the images were collected by fluorescence microscope (a). Scale bars = 10 μm. Female BALB/c mice were treated with TCS from 0 to 25 mg/kg for 90 min, western blot was used to assay the expressions of LC3 in livers (b) and spleens (c) respectively, compared with the control groups, * $p < 0.05$, ** $p < 0.01$, *** $p < 0.001$.
