# Supplementary material for: Triclosan Enhances the Clearing of Pathogenic Intracellular Salmonella or Candida albicans but Disturbs the Intestinal Microbiota through mTOR-Independent Autophagy
Source: Front Cell Infect Microbiol. 2018 Feb 21;8:49. doi: 10.3389/fcimb.2018.00049 (PMC5826388; doi:10.3389/fcimb.2018.00049)
Supplement: Supplementary file 2 [file Image2.PDF]

**Fig. S2 Further verification of the autophagic response induced by TCS.**

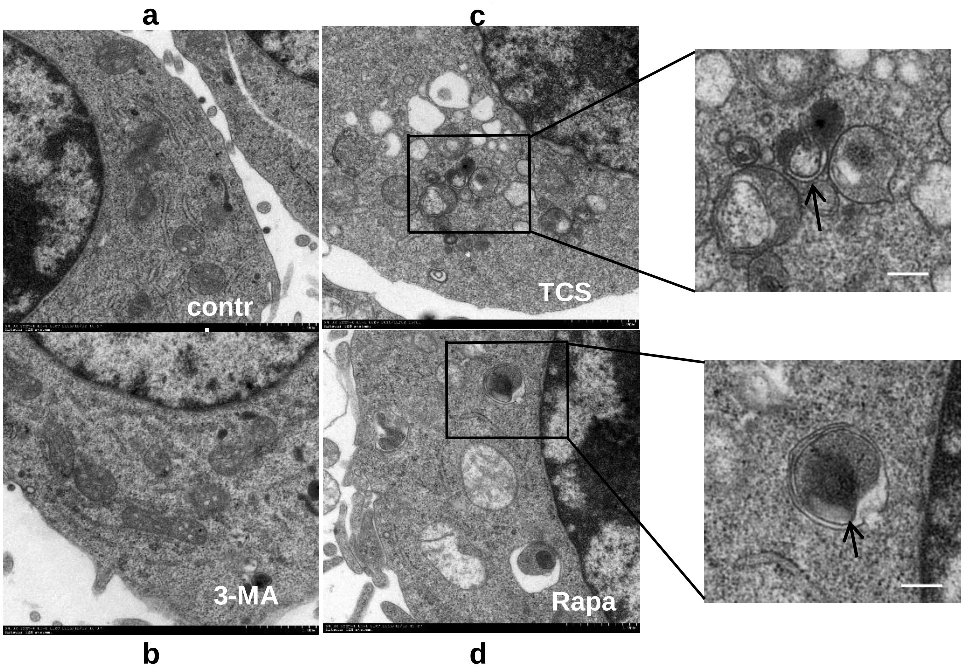


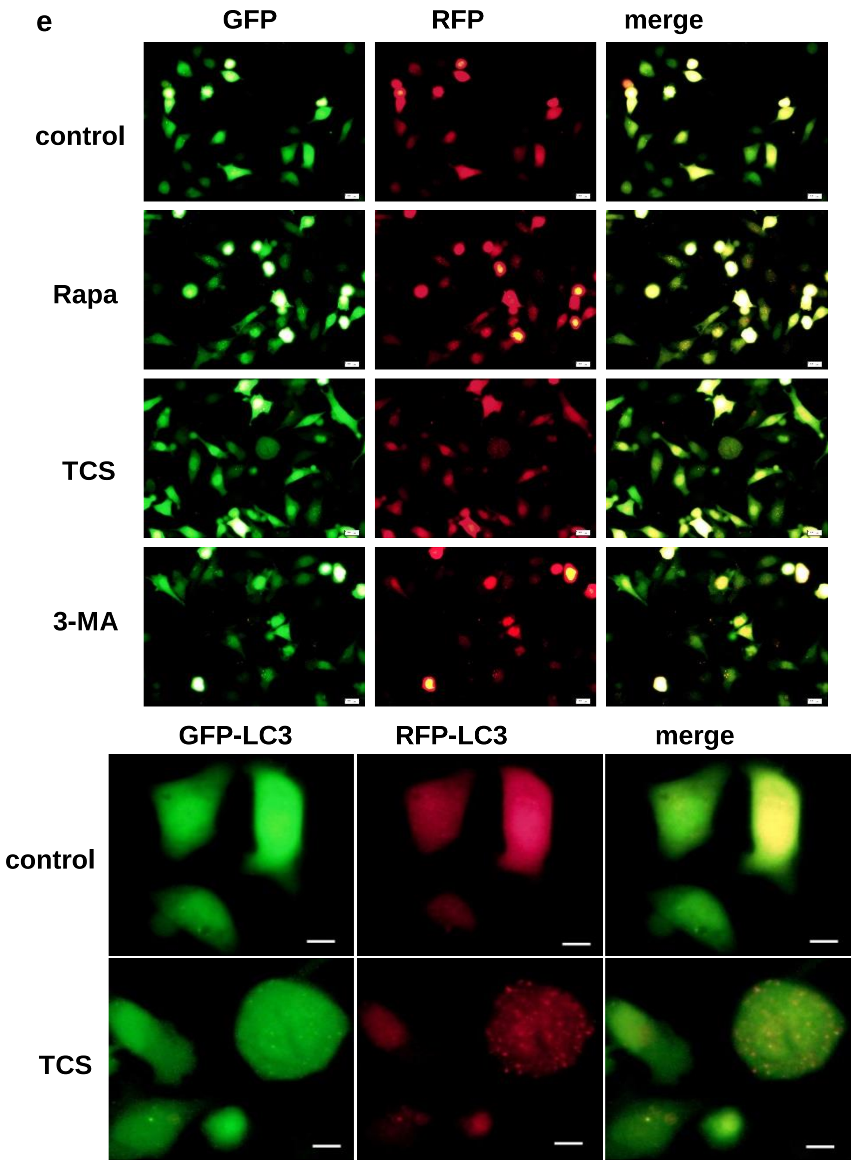


**Fig. S2 Further verification of the autophagic response induced by TCS.** RAW264.7 cells were incubated in 6 well plates at a density of 1 × $10^6$ cells/well. The cells treated with DMSO were control groups (a), the cells treated with Rapa (200 nM, 12 h) were positive controls (b), and 3-MA (5 mM, 180 min) treatment were negative controls (d). The cells treated with TCS (8 μM) for 180 min (c), the images were collected by TEM. The HeLa cells were transfected with RFP-GPF-LC3 plasmids and treated with TCS (8 μM) for 180 min, the images were obtained by fluorescence microscope (e), Scale bars = 10 μm.
