# Supplementary material for: Triclosan Enhances the Clearing of Pathogenic Intracellular Salmonella or Candida albicans but Disturbs the Intestinal Microbiota through mTOR-Independent Autophagy
Source: Front Cell Infect Microbiol. 2018 Feb 21;8:49. doi: 10.3389/fcimb.2018.00049 (PMC5826388; doi:10.3389/fcimb.2018.00049)
Supplement: Supplementary file 3 [file Image3.PDF]

Fig. S3 TCS stimulated autophagy through AMPK/ULK1 pathway instead of mTOR-dependent pathway in MΦ.

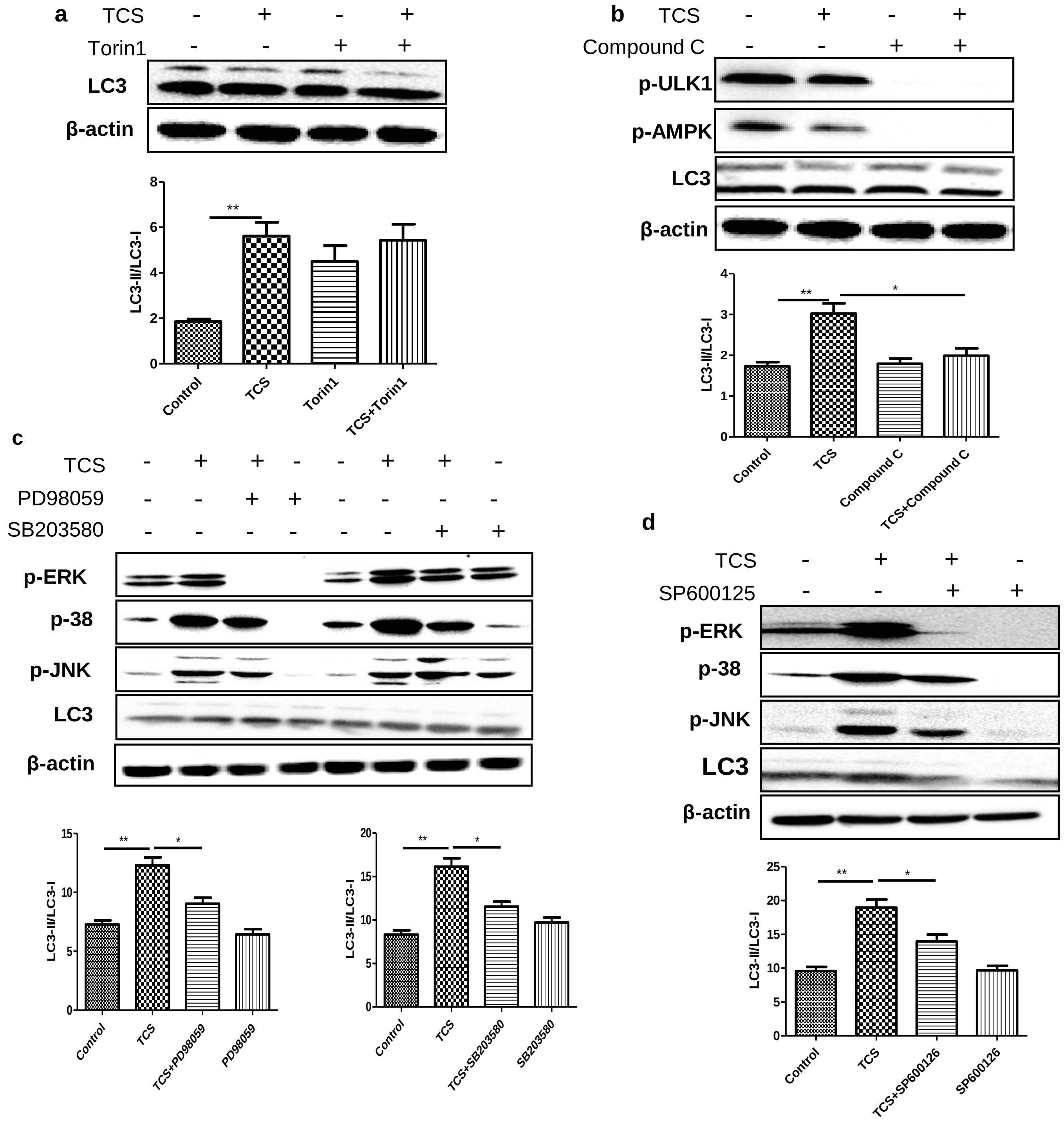

Fig. S3 TCS stimulated autophagy through AMPK/ULK1 and JNK/p38/ERK pathway instead of mTOR-dependent pathway in MΦ. The RAW264.7 cells were pretreated with 2 μM Torin 1 (a) or 5 mM Compound C (b) for 60 min, then were treated with TCS (8 μM) for 90 min, the expressions of LC3 and the levels of phosphorylation of AMPK and ULK1 were assayed by western blot. The cells were pretreated with PD98059 or SB203580 for 60 min, then were treated with TCS (8 μM) for 90 min, the expressions of LC3 and the levels of phosphorylation of JNK, ERK and p38 were assayed by western blot (c). The cells were pretreated with SP600125 for 60 min, then were treated with TCS (8 μM) for 90 min, the expressions of LC3 and the levels of phosphorylation of JNK, ERK and p38 were assayed by western blot (d). Compared to respective controls, * $p < 0.05$, ** $p < 0.01$, *** $p < 0.001$.
