# Supplementary material for: Triclosan Enhances the Clearing of Pathogenic Intracellular Salmonella or Candida albicans but Disturbs the Intestinal Microbiota through mTOR-Independent Autophagy
Source: Front Cell Infect Microbiol. 2018 Feb 21;8:49. doi: 10.3389/fcimb.2018.00049 (PMC5826388; doi:10.3389/fcimb.2018.00049)
Supplement: Supplementary file 4 [file Image4.PDF]

Fig. S4 TCS could improve the NDP52 expression and strengthen the colocalization among intracellular pathogens, NDP52 and LC3.

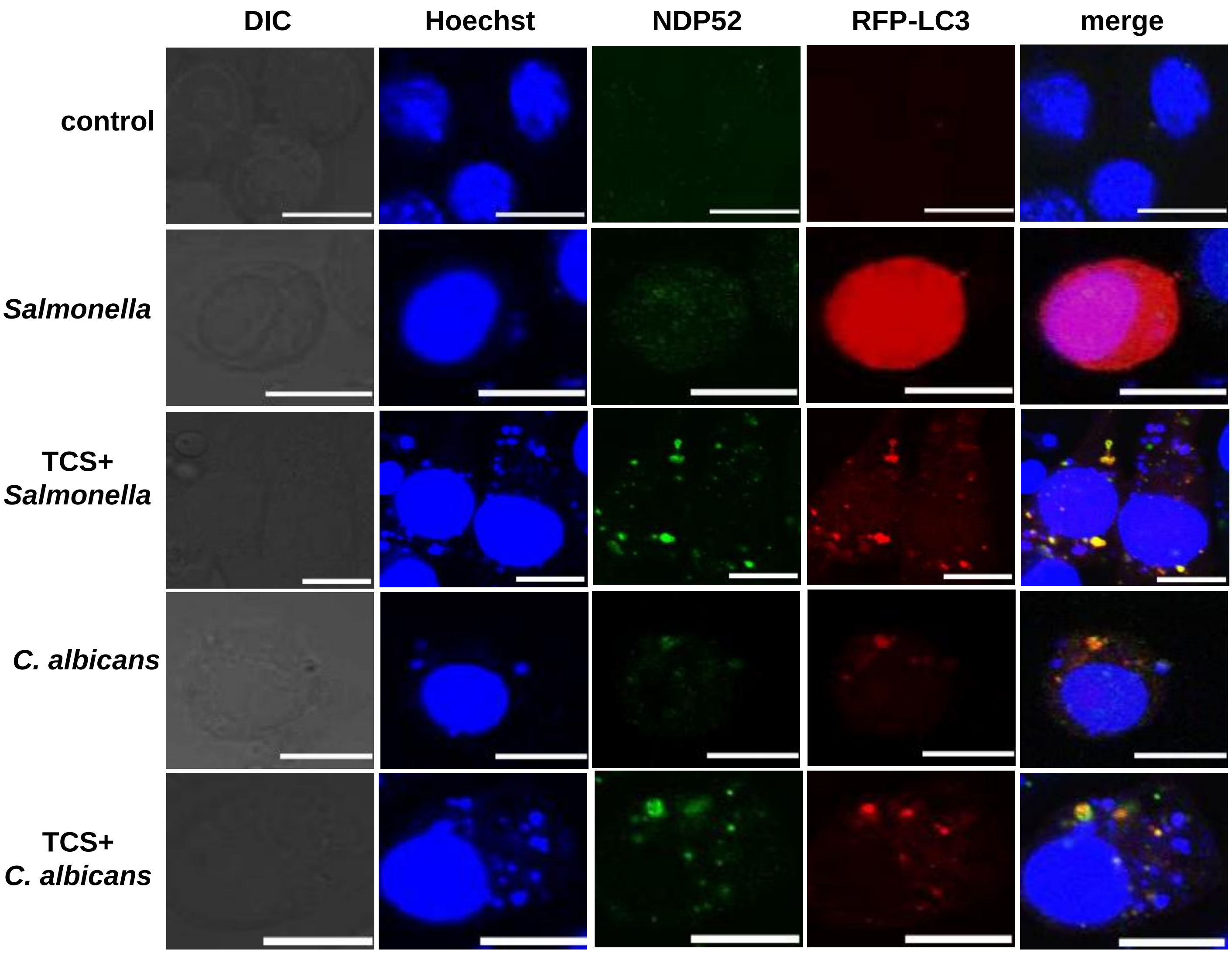

Fig. S4 TCS could improve the NDP52 expression and strengthen the colocalization among intracellular pathogens, NDP52 and LC3. The RAW264.7 cells were transfected with RFP-LC3 plasmids and incubated with or without pathogens for 45 min, the cells then treated with or without TCS (8 μM) for 90 min followed with immunofluorescent staining. NDP52 was marked by NDP52 antibody, the pathogens and nucleus were stained by Hoechst 33342. The results were collected by laser scanning confocal microscope. Scale bars = 10 μm
