# Supplementary material for: Triclosan Enhances the Clearing of Pathogenic Intracellular Salmonella or Candida albicans but Disturbs the Intestinal Microbiota through mTOR-Independent Autophagy
Source: Front Cell Infect Microbiol. 2018 Feb 21;8:49. doi: 10.3389/fcimb.2018.00049 (PMC5826388; doi:10.3389/fcimb.2018.00049)
Supplement: Supplementary file 5 [file Image5.PDF]

Fig. S5 The abilities of pathogens clearance of RAW264.7 cells were enhanced by TCS treatment.

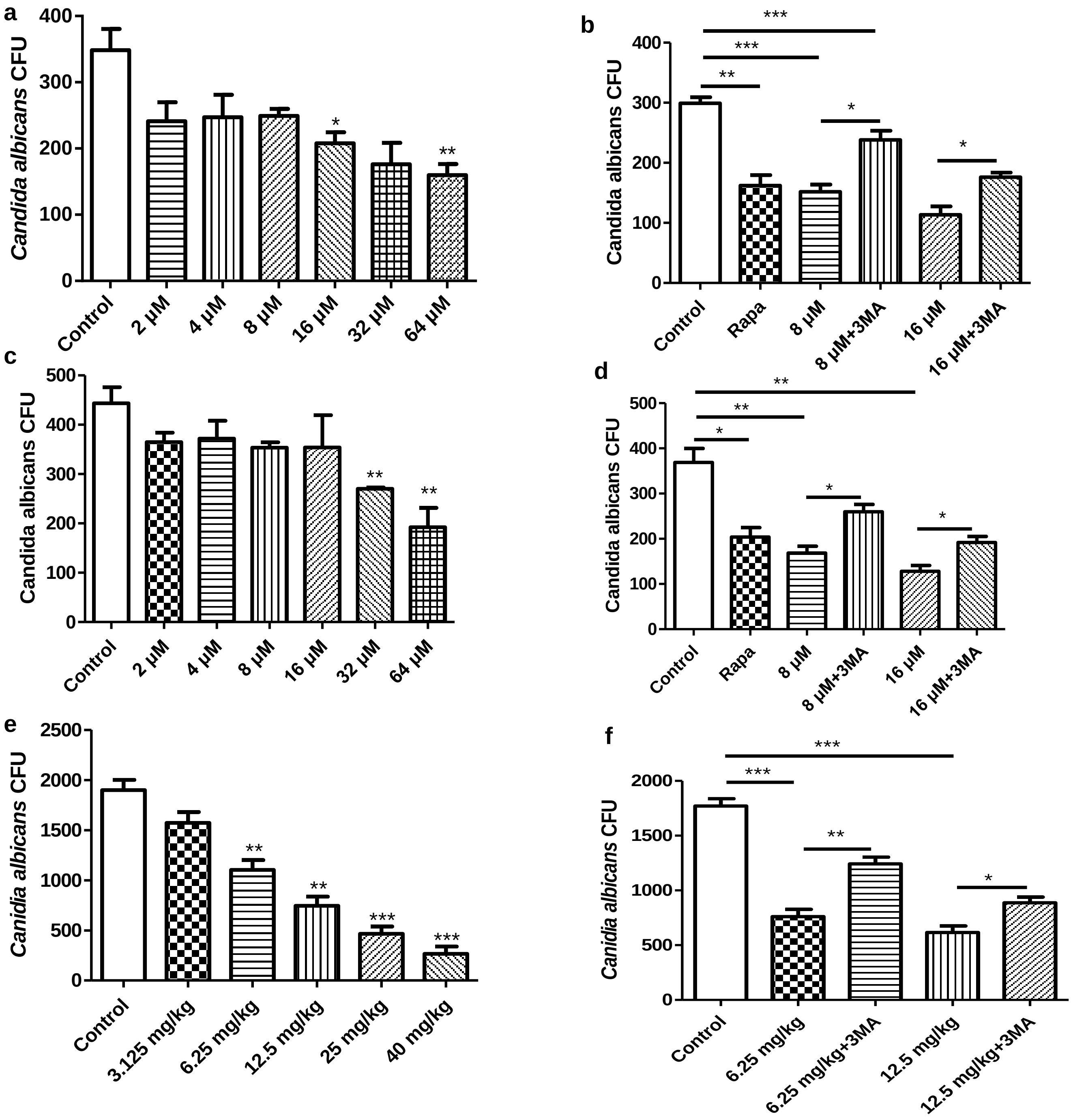

Fig. S5 The abilities of pathogens clearance were enhanced by TCS treatment *in vitro* and *in vivo.* RAW264.7 cells were incubated with TCS (0 to 64 μM, 90 min) (a) or Rapa (200 nM, 12 h), 3-MA (5 mM, 180 min) and TCS (8 μM, 90 min) (b) first and then infected with *C. albicans* at a MOI of 1 : 1. Colony counting was used to calculate the extracellular CFU. RAW264.7 cells were incubated with *C. albicans* at a MOI of 1 : 1 for 30 min. The cells then treated with TCS (0 to 64 μM, 90 min) (c) or Rapa (200 nM, 12 h), 3-MA (5 mM, 180 min) and TCS (8 μM, 90 min) (d) for 90 min. Colony counting was used to calculate the intracellular CFU. Female BALB/c mice were infected with *C. albicans* for three days, the mice were treated with or without 3-MA (24 mg/kg) first and treated with TCS orally three times a day for four days. The tongues were homogenized for colony counting (e, f). Compared to respective controls, * $p < 0.05$, ** $p < 0.01$, *** $p < 0.001$.
