# Supplementary material for: Triclosan Enhances the Clearing of Pathogenic Intracellular Salmonella or Candida albicans but Disturbs the Intestinal Microbiota through mTOR-Independent Autophagy
Source: Front Cell Infect Microbiol. 2018 Feb 21;8:49. doi: 10.3389/fcimb.2018.00049 (PMC5826388; doi:10.3389/fcimb.2018.00049)
Supplement: Supplementary file 6 [file Image6.PDF]

Fig. S6 The shifts of the bacterial community compositions detected by 16S rRNA gene sequencing.

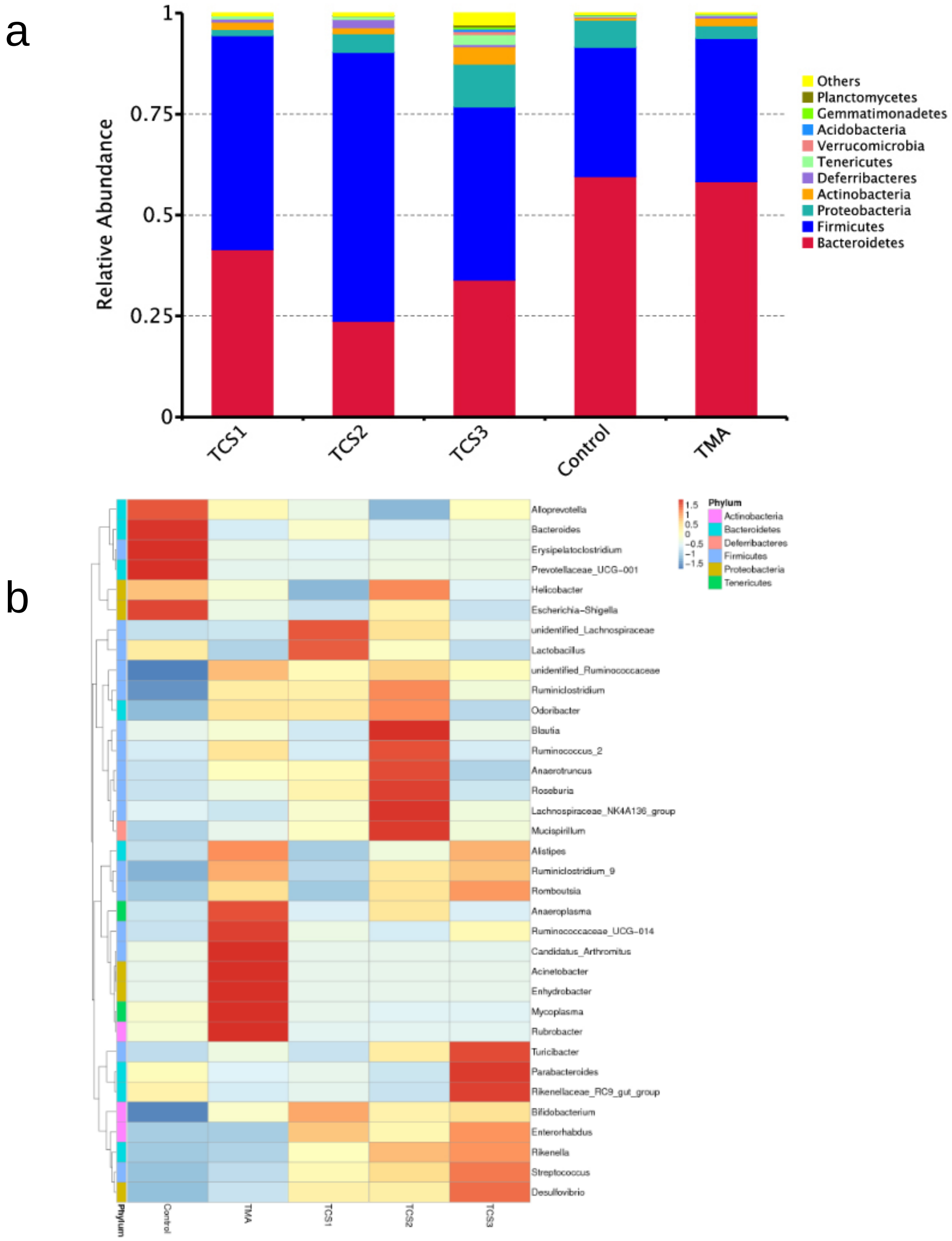

Fig. S6 The shifts of the bacterial community compositions detected by 16S rRNA gene sequencing. Female BALB/c mice were treated with vehicle (0.5% methylcellulose-0.1% Tween 80 in sterile water), TCS (1 mg/day, 2 mg/day, 3 mg/day), or TCS (3 mg/day) by oral ingestion of 15 mL and with 20µL of 3-MA (24 mg/kg) by an intraperitoneal injection for 30 days. From the 28th day to the30th day, mouse excrement was collected every morning. Relative abundance of the dominant bacterial in the intestinal communities. Each bar represents the relative abundance of each sample. Each color represents a particular bacterial phylum (a). Hierarchically clustered heat map analysis of the highly represented bacterial taxa (at the genus level) found in the intestinal communities. The relative percentages of the bacterial families are indicated by varying color intensities according to the legend at the top of the figure. The color key for the Z score indicates correspondence between blue-red coloring and standard deviations from the mean abundance of each bacterial taxon (b). TCS1- TCS 1 mg/day, TCS2- TCS 2 mg/day, TCS3- TCS 3 mg/day, Control- vehicle, TMA- TCS 3 mg/day and 3-MA 24 mg/kg.
